# Reservoir Computing Beyond Memory-Nonlinearity Trade-off

**DOI:** 10.1038/s41598-017-10257-6

**Published:** 2017-08-31

**Authors:** Masanobu Inubushi, Kazuyuki Yoshimura

**Affiliations:** 1 NTT Communication Science Laboratories, NTT Corporation, 3-1, Morinosato Wakamiya Atsugi-shi, Kanagawa, 243-0198 Japan; 2 Department of Information and Electronics, Graduate School of Engineering, Tottori University, 4-101 Koyama-Minami, Tottori, 680-8552 Japan

## Abstract

Reservoir computing is a brain-inspired machine learning framework that employs a signal-driven dynamical system, in particular harnessing common-signal-induced synchronization which is a widely observed nonlinear phenomenon. Basic understanding of a working principle in reservoir computing can be expected to shed light on how information is stored and processed in nonlinear dynamical systems, potentially leading to progress in a broad range of nonlinear sciences. As a first step toward this goal, from the viewpoint of nonlinear physics and information theory, we study the *memory*-*nonlinearity trade*-*off* uncovered by Dambre *et al*. (2012). Focusing on a variational equation, we clarify a dynamical mechanism behind the trade-off, which illustrates why nonlinear dynamics degrades memory stored in dynamical system in general. Moreover, based on the trade-off, we propose a *mixture* reservoir endowed with both linear and nonlinear dynamics and show that it improves the performance of information processing. Interestingly, for some tasks, significant improvements are observed by adding *a few* linear dynamics to the nonlinear dynamical system. By employing the echo state network model, the effect of the mixture reservoir is numerically verified for a simple function approximation task and for more complex tasks.

## Introduction

A variety of dynamical systems, including recurrent neural networks, soft material, and optoelectronic and quantum systems, exhibit *common*-*signal*-*induced synchronization* ^1–4^. These dynamical systems have a kind of reproducibility to a repeated input signal, and remarkably, can serve as a resource for information processing in principle. This framework is referred to as *reservoir computing* (*RC*)^5^ which was proposed originally in the research fields of machine learning^6^ (called Echo State Network) and computational neuroscience^7^ (called Liquid State Machine). Recent implementations of RC with the dynamical systems mentioned above have shown excellent performances in the processing of practical tasks such as time series forecasting and speech recognition^8–18^.

In the framework of RC, an input signal drives a dynamical system (called a reservoir), and we obtain a desired output through ‘careful’ observation of the transient states of the system. Specifically, as we describe below, a processed signal is obtained as a linearly weighted readout of the states. The linear weight is determined by using supervised machine learning (simply the least-squares method); therefore, the training procedure is computationally inexpensive, which allows us to utilize dynamical systems with a huge number of degrees of freedom for RC. Implementing RC with an optical system, which is of a large number of degrees of freedom, accomplishes fast information processing with low energy consumption^8–12, 14–17^, and it could potentially outperform conventional information processing technologies.

However, many aspects of RC remain unknown. For example, little is known about its working principle, and there are few theoretical answers to the following fundamental question: what characteristics of a dynamical system are crucial for high-performance information processing? Progress in theoretical research on RC could uncover not only a reservoir design principle, but also deepen our understanding of information processing in dynamical systems, in particular give an answer to the question, such as how dynamical systems store and process information, discussed in a community of nonlinear physics^19^. And also, it can be expected that some insights of working principles in RC may lead to progress in theoretical neuroscience.

We here study the roles of linear and nonlinear dynamics in RC which are still not fully understood. Focusing on the linear memory capacity, i.e., the ability to reconstruct the past input signal from the present reservoir state, Jaeger conducted pioneering studies on short-term linear memory capacity (*MC*) and showed theoretically that *MC* ≤ *N* for a reservoir with i.i.d. input signal, where *N* is the number of nodes (see Proposition 2 in ref. 20). Interestingly, they also showed that generically *MC* = *N* for a reservoir with a *linear* activation function and concluded with an open question: “*Are linear networks always optimal for large MC*”? Linear memory capacity increasing with the number of nodes linearly (i.e. *MC* ∝ *N*) is called *extensive* memory. Ganguli *et al*. introduced the total memory *J*_*tot*_ as an integrated Fisher memory curve that is independent of the input signal history and clarified that a certain type of the linear reservoir with a non-normal connection matrix can achieve the extensive memory: *J*_*tot*_ = *N*. On the other hand, they showed ${J}_{tot}\propto \sqrt{N}$ at best for a reservoir subject to saturating nonlinearity^21^ (see ref. 22 for the relation between the two memory capacities; *MC* and *J*_*tot*_). The best memory lifetime achieved by a nonlinear network reported so far is *O*(*N*/log*N*), i.e., nearly extensive, as rigorously estimated by Toyoizumi, where the nonlinearity is harnessed for the error-correcting^23^. In summary, extensive memory capacity can be realized by a *linear* reservoir, and memory capacity seems to be degraded by introducing nonlinearity into the reservoir dynamics.

Previous studies suggest that nonlinear dynamics might degrade the memory capacity; however, nonlinear dynamics is apparently important for RC. For instance, the so-called linearly inseparable problem^24^, which often appears in practical tasks, cannot be solved without the nonlinear transformation of the input signal. In other words, the nonlinear dynamics of the reservoir is essential for general information processing. Therefore, it can be expected that there exists some trade-off relation between linearity and nonlinearity in reservoir dynamics, which is required respectively for memory capacity and for the general information processing. In the seminal paper^25^, Dambre *et al*. introduced a computational capacity of a dynamical system which is a natural generalization of the linear memory capacity to the nonlinear one, by employing a complete orthonormal basis of a function space. Importantly, by using the computational capacity, they suggested that there exists the universal memory-nonlinearity trade-off relation, and moreover, demonstrated it numerically for some dynamical systems with different types of nonlinearity^25^. And also, other numerical studies have concluded that linear nodes are effective for linear memory capacity and the linear-like reservoir becomes optimal for a task requiring longer memory^26–28^.

In the present work, we introduce a simple task which has controllable memory and nonlinearity and clearly demonstrate the memory-nonlinearity trade-off on the task, using the echo state network (random network) model as a simple reservoir. Moreover, focusing on the variational equation from the viewpoint of information theory, we give a theoretical interpretation that reveals a dynamical mechanism illustrating how the nonlinear dynamics degrades memory as observed in the previous studies^25–27^. The theoretical interpretation will imply the trade-off is indeed *universal* in the sense that the memory degradation occurs independently of the form of the nonlinearity of the dynamical system.

What sort of dynamical system is preferable for the reservoir that realizes the universal (nonlinear) transformation of the input signal and possess the appropriate memory capacity? The pioneering works in this direction tackled to find the answer; Butcher *et al*.^29–31^ introduced RC with random static projection (R^2^SP) and Extreme Learning Machines with a time delay based on the discussion on the trade-off, and reported these architectures improves performance well for some tasks compared with the standard echo state network model. The trade-off suggests that coexistence of linearity and nonlinearity in RC will improve its performance. Actually, Vinckier *et al*.^15^ introduced a linear optical dynamics on a photonic chip with nonlinear readout and showed that it possesses a remarkably high (total) memory capacity, and interestingly, exhibits high-performances for the complex tasks.

Here, we consider the coexistence of linearity and nonlinearity in RC in a different way. Namely, we propose a novel reservoir structure endowed with both linear and nonlinear activation functions, which is referred to as *mixture reservoir*. We show that introducing the mixture reservoir improves the performance of information processing for a variety of simple tasks. Interestingly, for some tasks, significant improvements are observed by adding *a few* linear dynamics to the nonlinear dynamical system. Finally, we verify the effect of the mixture reservoir for more practical and complex tasks: time series forecasting of the Santa Fe Laser data set^32^ and the NARMA task.

## Results

### Formulation

We here consider the echo state network model, which uses a random recurrent neural network as a reservoir. Its time evolution is given by

1 $${x}_{i}(t+\mathrm{1)}=\varphi \,[{a}_{i}(t)]$$

2 $${a}_{i}(t)=g(\sum _{j=1}^{N}\,{J}_{ij}{x}_{j}(t)+\varepsilon s(t)),$$

where ${x}_{i}(t)\in {\mathbb{R}}\,(i=1,\ldots ,N)$ denotes the state variable of *i*th unit of the network at time $t\in {\mathbb{Z}}$, $s(t)\in {\mathbb{R}}$ is an input signal, and *g*, $\varepsilon \in {\mathbb{R}}$ are control parameters. The function $\varphi \,:{\mathbb{R}}\to {\mathbb{R}}$ is a so-called activation function. We use *N* = 100 and *ϕ*[*a*] = *a* or $\varphi [a]=\,\tanh \,a$ in the numerical experiments. Elements *J*_*ij*_ of the connection matrix are independently and identically drawn from the Gaussian distribution with mean zero and variance 1/*N*; ${J}_{ij}\sim {\mathscr{N}}\mathrm{(0},\,1/N)$. In the RC framework, we consider linear readout $\hat{y}(t)={\sum }_{j=1}^{N}\,{w}_{j}{x}_{j}(t)$, where ${\{{w}_{j}\}}_{j=1}^{N}$ is a set of readout weights. The goal of RC, in general, is to approximate the functional relation $y(t)=f(\{s(k{)\}}_{k=-\infty }^{t-1})=f(s(t-\mathrm{1)},\,s(t-\mathrm{2)},\,\ldots )$ by the linear readout $\hat{y}(t)$. Toward this end, utilizing a finite data set ${\{s(t),\,y(t)\}}_{t=1}^{T}$ (so-called training data), the readout weights are determined simply by minimizing the normalized mean square error, ${w}^{\ast }=\mathop{{\rm{\arg }}\,{\rm{\min }}}\limits_{w}\,E(w)$, where

3 $$E(w)=\frac{{\langle {(y(t)-\hat{y}(t))}^{2}\rangle }_{T}}{{\langle y{(t)}^{2}\rangle }_{T}}=\frac{{\langle {(y(t)-{\sum }_{j}{w}_{j}{x}_{j}(t))}^{2}\rangle }_{T}}{{\langle y{(t)}^{2}\rangle }_{T}},$$

where the brackets represent the time average ${\langle z(t)\rangle }_{T}=1/T\,{\sum }_{t=1}^{T}\,z(t)$ for any sequence ${\{z(t)\}}_{t=1}^{T}$. To evaluate the performance of RC, we use the generalization error *E*(*w* ^*^) throughout this paper, where its relation to the capacity *C* of the dynamical system defined by Dambre *et al*.^25^ is *C* = 1 − *E*(*w* ^*^). In the present formulation, the reservoir has two parameters, (*g*, *ε*), so the error depends on them; *E*(*w* ^*^|*g*,*ε*). Hereafter, the error ${\mathcal E}$ represents ${\mathcal E} \,:={{\rm{\min }}}_{(g,\varepsilon )\in P}\,E({w}^{\ast }|g,\varepsilon )$, where *P* is a region in the parameter space $P\,:=\{(g,\varepsilon )|g\in \mathrm{[0.1},\mathrm{3.0]},\varepsilon \in \mathrm{[0.2},\mathrm{6.0]}\}$. The minimum value of the error is obtained numerically by calculating the error in the parameter region *P* discretely with step size Δ*g* = 0.1, Δ*ε* = 0.2. We checked the main results of this paper are insensitive to the choice of the parameter space *P* and step sizes in the Supplemental Information.

### Common-signal-induced synchronization

**Figure 1:**
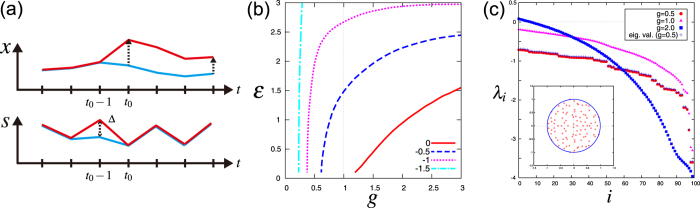
Conditional Lyapunov exponent. (**a**) The upper panel: schematic illustration of time evolution of the states *x*(*t*) and $\hat{x}(t)$ (the red and blue lines) in a state space and perturbation vectors (the black dot arrows). The lower panel: schematic illustration of the input signal sequences {*s*(*t*)}_*t*_ and ${\{\hat{s}(t)\}}_{t}$ for later use. (**b**) Contour lines of a (maximal) conditional Lyapunov exponent in parameter space (*g*, *ε*). The red line denotes the contour of the zero conditional Lyapunov exponent. (**c**) Conditional Lyapunov spectrum. For *ε* = 0.5, the red circles, purple triangles, and blue squares denote the conditional Lyapunov spectrum for *g* = 0.5, 1.0, and 2.0 respectively. The blue crosses denote ln |*σ*_*i*_(*gJ*)| where *σ*_*i*_(*gJ*) are eigenvalues of the connection matrix *gJ* with *g* = 0.5. The eigenvalues of *J* in a complex plane are shown in the subset (Circular Law of random matrix).

When employing a signal-driven dynamical system *x*(*t* + 1) = *T*(*x*(*t*), *s*(*t*)) as a reservoir, there is at least one necessary condition: the dynamical system has to exhibit *common*-*signal*-*induced synchronization*. Let us consider two different initial states *x*(*t*_0_) and $\hat{x}({t}_{0})$ (≠*x*(*t*_0_), see Fig. 1(a)). If these two states converge to the same state asymptotically under the action of the same dynamical system *T* and the *common* signal ${\{s(t)\}}_{t\ge {t}_{0}}$, i.e. $\Vert x(t)-\hat{x}(t)\Vert \to \mathrm{0\ (}t\to \infty )$, the signal-driven dynamical system *T* is said to exhibit common-signal-induced synchronization. This condition is also referred to as *echo state property* ^6^ or *consistency* ^33^. This condition means, if the transient state is discarded, the asymptotic state $x(t)\,(t\gg {t}_{0})$ depends not on the initial condition *x*(*t*_0_) but only on the sequence of the input signal ${\{s(t)\}}_{t\ge {t}_{0}}$. If the dynamical system (reservoir) does not satisfy this condition, different results will be obtained from the same input, depending on the initial condition of the reservoir.

A key quantity determining whether the reservoir satisfies these conditions is the *conditional Lyapunov exponent λ*({*s*(*t*)}) for a given signal sequence ${\{s(t)\}}_{t\in {\mathbb{Z}}}$. Let *δ*(*t*_0_) be an infinitesimally small difference in the initial states, $\delta ({t}_{0})=x({t}_{0})-\hat{x}({t}_{0})$. Then, the time evolution of the perturbation *δ*(*t*) is described by the variational equation *δ*(*t* + 1) = *DT*(*x*(*t*), *s*(*t*))*δ*(*t*), where *DT*(*x*(*t*), *s*(*t*)) is Jacobian matrix ${[DT(x(t),s(t))]}_{ij}\,:=\partial {T}_{i}/\partial {x}_{j}(x(t),s(t))$. The conditional Lyapunov exponent is given by $\lambda (\{s(t)\})={\mathrm{lim}}_{t\to \infty }\,\frac{1}{t}\,\mathrm{ln}\,\Vert \delta (t)\Vert$. Therefore, if *λ*({*s*(*t*)}) < 0 holds, the norm of the perturbation converges to zero asymptotically ||*δ*(*t*)|| ∝ *e* ^*λ*({*s*(*t*)})*t*^ → 0 (*t* → ∞), i.e., the negative conditional Lyapunov exponent implies the common-signal-induced synchronization.

In the above formulation, the variational equation of the dynamical system (2) is as follows:

4 $${\delta }_{i}(t+1)=g\sum _{j=1}^{N}\,{[DT(x(t),s(t))]}_{ij}{\delta }_{j}(t)\,\quad {\rm{w}}{\rm{h}}{\rm{e}}{\rm{r}}{\rm{e}}\quad {[DT(x(t),s(t))]}_{ij}\,:={\varphi }^{{\rm{^{\prime} }}}[{a}_{i}(t)]{J}_{ij}.$$

Figure 1(b) shows the contour line of the conditional Lyapunov exponent for the echo state network (2) with the activation function *ϕ*[*a*] = tanh *a* in the parameter space (*g*, *ε*). The input signal *s*(*t*) is independently and identically drawn from the uniform distribution in the interval (−1, 1), and we write the distribution as ${\mathscr{U}}\,(-1,\mathrm{1)}$. The red line represents *λ*({*s*(*t*)}) = 0, and hence, if the parameters are in the upper left region of this line, the dynamical system shows common-signal-induced synchronization and can be used for RC.

It is known that ref. 34, considering the deterministic case (i.e. *ε* = 0), the origin is a stable fixed point when *g* < 1 and chaotic behavior appears when *g* > 1. Moreover, it is also known that, the conditional Lyapunov exponent decreases when the input signal (noise) is added, i.e. the noise suppresses chaos. The numerical results are consistent with the theoretical results obtained by using the mean field approximation^34^. Figure 1(c) shows the conditional Lyapunov spectrum of the dynamical system (2) with the activation function *ϕ*[*a*] = tanh *a* for some parameter values (see Supplementary Information for the details).

### Memory-nonlinearity trade-off

First, we introduce a simple function approximation task. Although practical tasks such as time series forecasting are important, it is difficult to recognize in such complex tasks how an input signal should be transformed in a nonlinear way and how much memory capacity is required. Therefore, for a basic understanding of RC, we study the simple function approximation task first, which allows us to control the degree of the nonlinearity and the memory required in the tasks separately.

**Figure 2:**
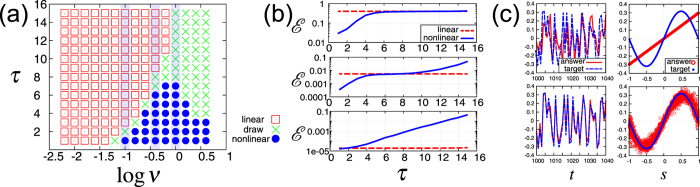
Memory-nonlinearity trade-off. (**a**) Diagram summarizing results of the direct comparison of the linear and nonlinear activation functions in the task parameter space $(\mathrm{log}\,\nu ,\tau )$. If ${ {\mathcal E} }_{L} < { {\mathcal E} }_{NL}$ (${ {\mathcal E} }_{L} > { {\mathcal E} }_{NL}$), we mark a red square (a blue circle). Each green cross represents draw, i.e. ${ {\mathcal E} }_{NL}/{ {\mathcal E} }_{L}\in \mathrm{(0.95},\,\mathrm{1.05)}$. (**b**) The error ${\mathcal E}$ versus *τ* for log *ν* = 0.0, −0.4, −1.0 from top to bottom. The blue lines (red broken lines) denote the error for the nonlinear (linear) reservoir. The left figure in (**c**) shows the time series of the target *y*(*t*) and the answer $\tilde{y}(t)$ for the task (log *ν*, *τ*) = (0, 2), and the right one shows function approximation plots where the horizontal axis is *s*(*t* − *τ*) and the vertical axis is *y*(*t*) and $\hat{y}(t)$. The upper (lower) figure corresponds to the results by the linear (nonlinear) reservoir.

The simple function approximation task requires computation *y*(*t*) = *f*(*s*(*t* − *τ*)), where *f* is a nonlinear function such as *f*(*x*) = sin *x*, tan *x*, and *x*(1 − *x* ^2^) and *s*(*t* − *τ*) is the input signal of *τ*-step before. For all results shown in this paper for the simple approximation tasks, the input signal *s*(*t*) is independently and identically drawn from the uniform distribution ${\mathscr{U}}(-1,\mathrm{1)}$. In Fig. 2, we show results in the case of *y*(*t*) = *f*(*s*(*t* − *τ*)) = sin (*νs*(*t* − *τ*)), where (*τ*, *ν*) are task parameters that control respectively the ‘depth’ of the required memory and ‘strength’ of the required nonlinearity.

We compare the linear function *ϕ*[*a*] = *a* and the nonlinear function *ϕ*[*a*] = tanh *a* to study the roles of the linearity and nonlinearity of the activation function in RC. We refer to the reservoir employing *ϕ*[*a*] = *a* (*ϕ*[*a*] = tanh *a*) as a linear (nonlinear) reservoir. As described in detail in the Supplemental Information, the linear reservoir can be interpreted as *ε* → 0 limit of the nonlinear reservoir.

Figure 2(a) shows a diagram summarizing the results of the direct comparison of the two activation functions in the task parameter space. For some parameters (*τ*, *ν*), if the error with the linear reservoir, ${ {\mathcal E} }_{L}$, is lower than that with the nonlinear reservoir, ${ {\mathcal E} }_{NL}$, i.e., ${ {\mathcal E} }_{L} < { {\mathcal E} }_{NL}$, we mark a red square at (*τ*, *ν*) in the diagram. Otherwise, if ${ {\mathcal E} }_{L} > { {\mathcal E} }_{NL}$, we mark a blue circle. The green crosses represent draw, i.e., ${ {\mathcal E} }_{NL}/{ {\mathcal E} }_{L}\in \mathrm{(0.95},\mathrm{1.05)}$. The errors ${\mathcal E}$ for log *ν* = 0.0, −0.4, −1.0 along *τ* (blue strips depicted in Fig. 2(a)) are shown in Fig. 2(b) from top to bottom. As a reference, typical examples of time series and ‘function approximation plots’, which illustrate how the function approximation is performed, are depicted in Fig. 2(c). While these results are obtained by employing a particular realization of random matrix *J* and a particular task *f*(*x*) = sin *x*, we confirmed that qualitatively the same results are obtained by employing other realizations of *J* and other tasks *f*(*x*) = tan *x* and *x*(1 − *x* ^2^).

These results indicate that, if the task requires ‘strong’ nonlinear transformation with ‘short’ memory ($\mathrm{log}\,\nu \mathop{ > }\limits_{ \tilde {}}-0.5,\tau \mathop{ < }\limits_{ \tilde {}}4$), the nonlinear reservoir outperforms the linear one. If the task requires ‘long’ memory with ‘weak’ nonlinear transformation ($\mathrm{log}\,\nu \mathop{ < }\limits_{ \tilde {}}-1.0,\tau \mathop{ > }\limits_{ \tilde {}}4$), the linear reservoir outperforms the nonlinear one. The linear dynamics is suitable for tasks requiring memory, although the linear dynamics cannot perform nonlinear transformation. On the other hand, the nonlinear dynamics is suitable for the tasks requiring nonlinear transformation, although the nonlinearity of the dynamics seems to degrade the linear memory capacity. In this sense, the above direct comparison clearly shows the memory-nonlinearity trade-off, which is consistent with previous studies^25, 27^.

### Why nonlinear dynamics degrades memory

The nonlinearity of the dynamics seems to degrade memory. We show that it can be interpreted by employing the variational equation with the viewpoint of information theory. First, we introduce two sequences of the input signals, {*s*(*t*)}_*t*_ and ${\{\hat{s}(t)\}}_{t}$, and assume that they are the same except for *t* = *t*_0_ − 1, i.e. ${\{\hat{s}(t)\}}_{t}={\{s(t)\}}_{t}$ for *t* ≠ *t*_0_ − 1 and $\hat{s}({t}_{0}-\mathrm{1)}=s({t}_{0}-\mathrm{1)}+{\rm{\Delta }}$, where Δ represents a small difference in the two sequences (see Fig. 1(a)). For simplicity, let us consider the case of *N* = 1 (see Supplementary Information for general dimensional case (*N* ≥ 1)). The difference in the input signal Δ leads to a difference in states; the state driven by the input sequence ${\{\hat{s}(t)\}}_{t}$ is described by $\hat{x}({t}_{0})=\varphi [g(Jx({t}_{0}-\mathrm{1)}+\varepsilon \hat{s}({t}_{0}-\mathrm{1))]}=x({t}_{0})+{\delta }_{0}$ in the range of linear approximation, where ${\delta }_{0}\,:=g\varepsilon \varphi ^{\prime} [a({t}_{0}-\mathrm{1)]}{\rm{\Delta }}$ and *x*(*t*_0_) is the state driven by {*s*(*t*)}_*t*_. The sequence ${\{{\delta }_{k}\}}_{k\mathrm{=0}}^{n}=({\delta }_{0},{\delta }_{1},\ldots ,{\delta }_{n})$ represents the difference between two orbits *x*(*t*_0_ + *k*) and $\hat{x}({t}_{0}+k)$, i.e., ${\delta }_{k}=\hat{x}({t}_{0}+k)-x({t}_{0}+k)\,(k\ge \mathrm{0)}$.

Let us consider the ability to reconstruct the initial difference *δ*_0_ from the later difference *δ*_*n*_ as *memory*. If there exists some relation between *δ*_0_ and *δ*_*n*_ (e.g., they are functionally dependent on each other), we could reconstruct the initial difference *δ*_0_ from *δ*_*n*_. In other words, it is potentially possible to readout some information about the past difference in the input sequences from the present reservoir state. In that case, it can be interpreted that the reservoir stores memory. On the other hand, if there is no relation between *δ*_0_ and *δ*_*n*_ (e.g., they are independent of each other), we cannot reconstruct the initial difference *δ*_0_ from the later difference *δ*_*n*_. In other words, we cannot readout any information about the past difference in the input sequences from the present reservoir state. In that case, it can be interpreted that the reservoir forgets memory.

The relation between *δ*_0_ and *δ*_*n*_ is given by the variational equation,

5 $${\delta }_{n}={(gJ)}^{n}\,({{\rm{\Pi }}}_{j=0}^{n-1}\,{\varphi }^{{\rm{^{\prime} }}}\,[a({t}_{0}+j)])\,{\delta }_{0}\,\quad \quad (n\ge 1),$$

in the range of linear approximation. In the linear reservoir case, we obtain a deterministic relation *δ*_*n*_ = (*gJ*)^*n*^ *δ*_0_ since *ϕ*′[*x*] = 1. Therefore, there exists a strong relation between *δ*_0_ and *δ*_*n*_, which is suitable for storing memory. In the nonlinear reservoir case, the product term ${{\rm{\Pi }}}_{j=0}^{n-1}\,\varphi ^{\prime} \,[a({t}_{0}+j)]$, which depends on the sequence ${\{x({t}_{0}+j)\}}_{j\mathrm{=0}}^{n-1}$ and ${\{s({t}_{0}+j)\}}_{j\mathrm{=0}}^{n-1}$, is a kind of ‘noise’ in view of preserving the information of *δ*_0_, because the product term does not correlate with *δ*_0_. Hence, the product term due to the nonlinearity always weakens the relation between *δ*_0_ and *δ*_*n*_, implying that introducing nonlinearity degrades memory. In brief, it can be interpreted that the nonlinear dynamics degrades memory, while the linear dynamics preserves it.

To study the above statement more quantitatively, we measure the strength of the relation using the mutual information *I*(*δ*_0_; *δ*_*n*_). Then, simply by using the fundamental inequality (*data*-*processing inequality*) in information theory, we can show $I({\delta }_{0};{\delta }_{n}^{L})\ge I({\delta }_{0};{\delta }_{n}^{NL})$ below, where ${\delta }_{n}^{NL}\,:={(gJ)}^{n}\,({{\rm{\Pi }}}_{j\mathrm{=0}}^{n-1}\,\varphi ^{\prime} \,[a({t}_{0}+j)]){\delta }_{0}$ and ${\delta }_{n}^{L}\,:={(gJ)}^{n}{\delta }_{0}$. To define the mutual information, we introduce a joint probability density function ${\tilde{p}}_{{x}_{0}}(\{{\delta }_{i}{\}}_{i\mathrm{=0}}^{n},\{{s}_{i}{\}}_{i\mathrm{=0}}^{n-1})$. Here, *δ*_0_ denotes the random perturbation at the initial point *x*_0_ in the state space of the signal-driven dynamical system *x*_*k*+1_ = *T*(*x*_*k*_, *s*_*k*_). Let us consider that *δ*_0_ is drawn from *p*(*δ*_0_) independently of the initial point *x*_0_. We write the perturbation vector at *x*_*n*_ as *δ*_*n*_. The mutual information can be defined by ${I}_{{x}_{0}}\,({\delta }_{0},{\delta }_{n})={h}_{{x}_{0}}\,({\delta }_{0})-{h}_{{x}_{0}}\,({\delta }_{0}|{\delta }_{n})$ by using the marginalized probability density function

6 $$\begin{array}{ccc}{p}_{{x}_{0}}({\delta }_{n},{\delta }_{0}) & := & \int \,{\mathop{p}\limits^{ \sim }}_{{x}_{0}}(\{{\delta }_{i}{\}}_{i=0}^{n},\{{s}_{i}{\}}_{i=0}^{n-1})d{\delta }_{1}\cdots d{\delta }_{n-1}d{s}_{0}\cdots d{s}_{n-1}\\  & = & p({\delta }_{0})\,\int \,{{\rm{\Pi }}}_{i=1}^{n-1}\,p({s}_{i}){{\rm{\Pi }}}_{i=1}^{n}p({\delta }_{i}|{\delta }_{i-1},{x}_{0},\{{s}_{j}{\}}_{j=0}^{i-1})\\  &  & \times d{\delta }_{1}\cdots d{\delta }_{n-1}d{s}_{0}\cdots d{s}_{n-1},\end{array}$$

where ${h}_{{x}_{0}}$ represents differential entropy defined by ${p}_{{x}_{0}}({\delta }_{n},{\delta }_{0})$.

The inequality implying ‘nonlinearity degrades memory’ can be shown by simply employing the *data*-*processing inequality* (Theorem 2.8.1 in ref. 35). Let $X\sim p({\delta }_{0})$ and *Y* = (*gJ*)^*n*^ *X* be random variables. Finally, we introduce $Z=g(Y)\,:={{\rm{\Pi }}}_{j=0}^{n-1}\,\varphi ^{\prime} \,[a({t}_{0}+j)]\cdot Y$. Then, *X* → *Y* → *Z* can be considered as a Markov chain. The data processing inequality implies *I*(*X*; *Y*) ≥ *I*(*X*; *Z*), and from the variational equation, ${I}_{{x}_{0}}\,({\delta }_{0};{\delta }_{n}^{L})=I\,(X;Y)$ and ${I}_{{x}_{0}}\,({\delta }_{0};{\delta }_{n}^{NL})=I\,(X;Z)$. Therefore, we obtain ${I}_{{x}_{0}}\,({\delta }_{0};{\delta }_{n}^{L})\ge {I}_{{x}_{0}}\,({\delta }_{0};{\delta }_{n}^{NL})$ and this inequality holds for each *x*_0_ in the state space, i.e., nonlinearity degrades memory.

Note that the above argument is general in two senses. First, it does not assume any particular function form of the map *T* defining dynamical system *x*(*t* + 1) = *T*(*x*(*t*), *s*(*t*)). Therefore, we conclude that introducing *any form* of nonlinearity in the reservoir dynamics degrades memory, which suggests a positive resolution of the ‘Jaeger conjecture’^20^: linear networks are always optimal for large memory capacity. Second, the above argument does not assume linear readout, which is specific to RC. Thus, the above statement, *nonlinearity degrades memory*, holds for general signal-driven dynamical systems.

### Beyond the trade-off

We showed the memory-nonlinearity trade-off in our numerical experiment, and gave the dynamical mechanism behind the trade-off. With this trade-off, it is natural to use both linear and nonlinear activation functions with an expectation of storing memory by linear dynamics and realizing general transformation by nonlinear dynamics. We show numerically that a reservoir endowed with both linear and nonlinear activation functions, hereafter referred to as a *mixture reservoir*, is superior to the linear or nonlinear reservoir. Here the effect of the mixture reservoir is demonstrated for the simple function approximation task *y*(*t*) = sin (*νs*(*t* − *τ*)).

We extend the standard echo state network as follows:

7 $${x}_{i}(t+1)={\varphi }_{i}[{a}_{i}(t)]\,\quad {\rm{w}}{\rm{h}}{\rm{e}}{\rm{r}}{\rm{e}}\quad \,{\varphi }_{i}[x]=\{\begin{array}{cc}x\quad \, & (i\in {V}_{L})\\ \tanh \,[x]\quad  & (i\in {V}_{NL}),\end{array}$$

where *a*_*i*_(*t*) is the same as in the equation (2). *V*_*L*_ is an index set corresponding to the set of nodes utilizing linear activation function (linear nodes): *V*_*L*_ = {1, …, *N*_*p*_}, *V*_*NL*_ is that utilizing nonlinear activation function (nonlinear nodes): *V*_*L*_ = {*N*_*p*_ + 1, …, *N*}. Let *p* be ‘mixture rate’ of the linear and nonlinear reservoir: *p* = 1 − *N*_*p*_/*N*, i.e., *p* = 0 (resp. *p* = 1) means all of the activation functions are linear (resp. nonlinear), and 0 < *p* < 1 means the reservoir consists of the linear and nonlinear activation functions. Here, we use again the random matrix *J*_*ij*_ as the connection matrix, and therefore, the mixture reservoir we introduce is the network with linear and nonlinear nodes that are randomly coupled. Throughout this paper, for each fixed mixture rate *p*, the error ${\mathcal E}$ is obtained with the optimal parameter (*g*, *ε*) in the parameter space *P* as described in the Formulation.

**Figure 3:**
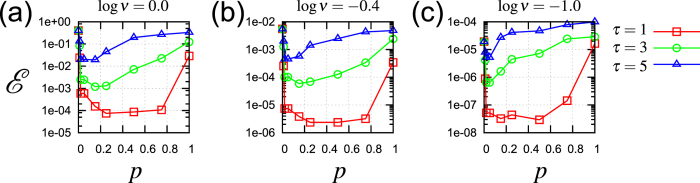
Performance improvement by the mixture reservoir. The error ${\mathcal E}$ versus the mixture rate *p*. The task parameters are (**a**) log *ν* = 0.0, (**b**) log *ν* = −0.4, and (**c**) log *ν* = −1.0. The red squares, green circles, and blue triangles correspond to the task parameters *τ* = 1, 3, 5, respectively. (See Supplementary Information for the enlarged figures around *p* = 0).

Figure 3 shows the approximation error ${\mathcal E}$ versus the mixture rate *p* for some tasks. As an example of a task requiring nonlinear transformation, the error in the case of log *ν* = 0 is depicted in Fig. 3(a) with *τ* = 1, 3, 5. As seen in the above results (Fig. 2), the nonlinear reservoir outperforms the linear one for this task, and, correspondingly, the error ${\mathcal E}$ at *p* = 1 is less than that at *p* = 0 in Fig. 3(a) (see Supplementary Information for the enlarged view of these figures). Furthermore, the error ${\mathcal E}$ at *p* ∈ (0, 1) is less than those two cases, i.e., the mixture reservoir outperforms both the linear and nonlinear reservoir. Note that the errors of the mixture reservoir at *p* = 0.1 are considerably less than those of the linear reservoir (*p* = 0.0). Moreover, for the case of *τ* = 1, the errors of the mixture reservoir at *p* = 0.8 are considerably less than those of the nonlinear reservoir (*p* = 1.0). It is interesting that introducing only a few nonlinear (linear) nodes to the linear (nonlinear) reservoir improves its performance remarkably. For other tasks as well, the same remarkably improvements can be found qualitatively (see Fig. 3(b,c)).

**Figure 4:**
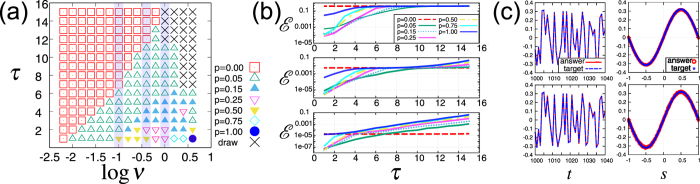
The mixture reservoir is effective for a broad region in the task parameter space. (**a**) Diagram summarizing results of the optimal mixture rate *p*_opt._(*ν*, *τ*). The different symbols represent the different optimal mixture rates in *p* ∈ {0.00, 0.05, 0.15, 0.25, 0.50, 0.75, 1.00}. (**b**) The error ${\mathcal E}$ versus *τ* for log *ν* = 0.0, −0.4, −1.0 from top to bottom. The different lines represent the different mixture rates as in (**a**). (**c**) Time series of the target *y*(*t*) and the answer $\tilde{y}(t)$ for the task (log *ν*, *τ*) = (0, 2) (left) as shown in Fig. 2(c). The function approximation plots (right). The upper (lower) panel corresponds to the case for the mixture rate *p* = 0.75 (*p* = 0.25).

An optimal mixture rate depends on the task, i.e., ${p}_{{\rm{opt}}.}(\nu ,\tau ):=\mathop{{\rm{\arg }}\,{\rm{\min }}}\limits_{p\in \mathrm{[0},\mathrm{1]}}\, {\mathcal E} (p|\nu ,\tau )$, where ${\mathcal E} \,(p|\nu ,\tau )$ denotes the error with a mixture rate *p* for a given task (*ν*,*τ*). To study this dependency, we show the optimal mixture rates in the diagram in the Fig. 4(a). As in the diagram in Fig. 2(a), for a set of given task parameters (*ν*, *τ*), we indicate the optimal mixture rate *p*_opt._(*ν*, *τ*) with different symbols, where the minimal value is numerically found in the set *p* ∈ {0.00, 0.05, 0.15, 0.25, 0.50, 0.75, 1.00}. The crosses represent draw again, i.e. ${min}_{p}\,{\mathscr{E}}(p|\nu ,\tau )/{max}_{p}\,{\mathscr{E}}(p|\nu ,\tau )\in (0.95,1.00]$. As in Fig. 2(b,c), the error ${\mathcal E}$, time series, and function approximation plots are shown in Fig. 4(b,c).

The diagram indicates that the optimal mixture rate depends on the task gradually, and, importantly, the mixture reservoir (0 < *p* < 1) outperforms the linear and nonlinear reservoir (*p* = 0, 1) over a broad region in the task parameter space.

### More complex tasks

The simple function approximation task *y*(*t*) = sin (*νs*(*t* − *τ*)) allows us to explicitly decompose the degree of the nonlinearity and memory required for the task. However, practically important tasks such as time series prediction are much more complicated than the tasks employed above. Here, we study the effect of introducing the mixture reservoir for two more practical tasks: time series forecasting of the Santa Fe Laser data set^32, 36^ and the NARMA task. These tasks are frequently used in the RC studies^26–28, 36^ to assess the performance of the reservoir.

**Figure 5:**
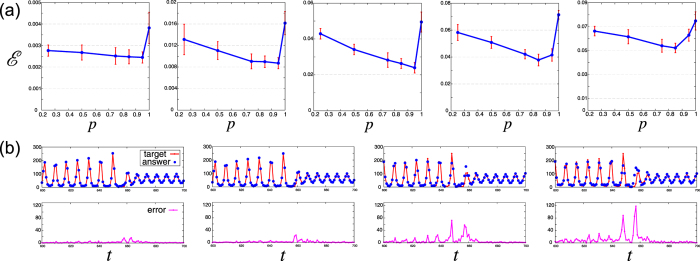
Time series forecasting of the Santa Fe Laser data set. (**a**) Error ${\mathcal E}$ versus the mixture rate *p* for the *k*-step ahead prediction with *k* = 1, 2, 3, 4, 5 from left to right. The error bar represents the standard deviation of the prediction error for 10 different connection matrices *J*. (**b**) The upper panels show the time series of the target *y*(*t*) (i.e., the Santa Fe Laser data set) and the answer $\tilde{y}(t)$ (i.e., the predicted value), corresponding to the red line and blue dots, respectively. The left two panels are the time series for the one-step ahead prediction, with the mixture rate *p* = 0.95 (left) and *p* = 1.0 (right). The right two panels are the time series for the three-step ahead prediction, with the mixture rate *p* = 0.95 (left) and *p* = 1.0 (right). The lower panels show their error values corresponding to the upper panels.

The Santa Fe Laser data set is a time series {*y*(*t*)} obtained from chaotic laser experiments. Given the past data $(\ldots ,y(t-\mathrm{2),}y(t-\mathrm{1),}y(t))$, the task is to predict future values *y*(*t* + *k*) (*k* ≥ 1), which is referred to as a *k*-step ahead prediction. We show the prediction errors for the *k* = 1, 2, 3, 4, 5 versus the mixture rate *p*(∈$[0.25,\,1.0]$) in Fig. 5(a), where the error with *p* = 0 is not depicted because of its large value, i.e., the linear reservoir does not work at all for this task. The time series of the original data *y*(*t*) and the predicted data $\hat{y}(t)$ are depicted in Fig. 5(b). In each case of *k*, introducing the mixture reservoir suppresses the error. Let us define the error suppression rate as $R:= {\mathcal E} (p={p}_{{\rm{opt}}.})/ {\mathcal E} (p=\mathrm{1)}$ for a fixed task. Then, the error suppression rate *R* attains its minimum for the three-step ahead prediction ($R\simeq 0.5$), while *R* > 0.5 for the one-step and five-step ahead predictions. The optimal mixture rate *p*_opt._ depends on *k* in this task as well, and, interestingly, *p*_opt._(*k*) decreases with increasing *k*, i.e., to accomplish a prediction of a more distant future, the reservoir needs more linear dynamics.

**Figure 6:**
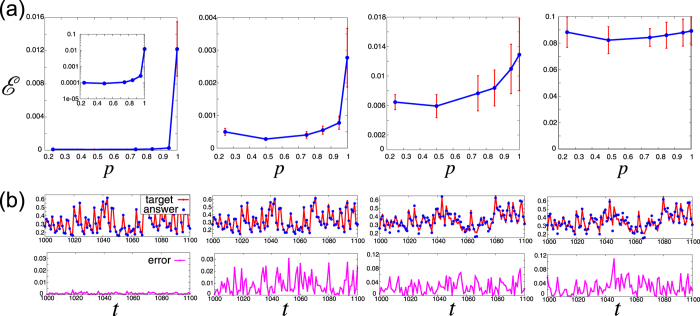
NARMA task. (**a**) Error ${\mathcal E}$ versus the mixture rate *p* for the parameters *m* = 1, 2, 5, 10 from left to right. The error bar represents the standard deviation of the prediction error for 10 different connection matrices *J*. The inset in the left panel is its semi-log plot. (**b**) The upper panels show the time series of the target *y*(*t*) (i.e., the NARMA system) and the answer $\tilde{y}(t)$ (i.e., the emulated value), corresponding to the red line and blue dots respectively. The left two panels are the time series for the NARMA1 task (*m* = 1), with the mixture rate *p* = 0.5 (left) and *p* = 1.0 (right). The right two panels are the time series for the NARMA10 task (*m* = 10), with the mixture rate *p* = 0.5 (left) and *p* = 1.0 (right). The lower panels show their error values corresponding to the upper panels.

The NARMA task is to emulate a signal-driven dynamical system with a highly nonlinear auto-regressive moving average as follows: $y(t)=\alpha y(t-\mathrm{1)}+\beta y(t-\mathrm{1)}\,{\sum }_{i=1}^{m}\,y(t-i)$ + $\gamma s(t-m)s(t)+\delta$, where *α* = 0.3, *β* = 0.05, *γ* = 1.5, and *δ* = 0.1. Note that the parameter *m* changes simultaneously both the required memory and nonlinearity. The signal *s*(*t*) is independently and identically drawn from the uniform distribution ${\mathscr{U}}\mathrm{[0},0.5]$, which drives both the NARMA system and the reservoir. Figure 6(a) shows the error in the emulation of the NARMA system with parameters *m* = 1, 2, 5, 10 by the mixture reservoir *p* ∈ $[0.25,\,1.0]$. Correspondingly, typical time series are depicted in Fig. 6(b). For the task *m* = 10, the errors with several mixture rates *p* are almost the same (or the mixture reservoir with *p* = 0.5 is slightly better than the others). However, for the tasks *m* = 1, 2, 5, the error is clearly reduced by introducing the mixture reservoirs. Furthermore, the smaller parameter *m* is, the more effective the mixture reservoir becomes, e.g., the error suppression rate $R\simeq 0.5$ when *m* = 5, and, moreover, $R\simeq 0.008$ when *m* = 1.

## Discussion

In the present work, we numerically demonstrated the memory-nonlinearity trade-off for the echo state network model. Namely, the linear dynamics is suitable for storing memory but useless for nonlinear transformation, while the nonlinear dynamics is suitable for nonlinear transformation but degrades memory.

We have uncovered the dynamical mechanism behind the memory-nonlinearity trade-off, using the variational equation from the viewpoint of information theory. The mechanism describes how the nonlinear dynamics degrades memory and the linear dynamics preserves it. In terms of information theory, storing memory with the nonlinear (resp. linear) dynamics corresponds to transferring message in a noisy (resp. noiseless) communication channel. The above theoretical interpretation assumes neither the function form of nonlinearity in the reservoir dynamics nor the linear readout. Hence, we conclude that, as a property of general signal-driven dynamical systems, introducing nonlinearity in the dynamics *always* degrades memory (Jaeger conjecture^20^).

On the basis of the memory-nonlinearity trade-off, we proposed the mixture reservoir, which is endowed with both linear and nonlinear dynamics. We numerically showed that it reduces function approximation errors effectively. Moreover, the observation shows that adding ‘a pinch of linearity’ considerably improves the performance of the nonlinear reservoir. This conclusion may be valuable for physical implementation of RC, since nonlinear dynamical systems are often used for the reservoir. While the both effects of adding ‘a pinch of linearity’ and ‘a pinch of nonlinearity’ to the RC performance are numerically observed for some tasks, the magnitudes of the effects may depend on how much nonlinearity or memory the task requires. For instance, in Fig. 3(a), adding ‘a pinch of linearity’ is not effective for *τ* = 5. It can be interpreted as the task *τ* = 5 requires ‘deep’ memory, and thus, adding a large amount of linearity is needed.

Finally, we verified the effect of the mixture reservoir in more practical and complex tasks, time series forecasting of the Santa Fe Laser data set^32^ and the NARMA task. It is interesting to note that the optimal mixture rate *p* changes depending on the tasks: in the Santa Fe time series forecasting task ${p}_{{\rm{opt}}.}\simeq 0.9$; on the other hand, in the NARMA task ${p}_{{\rm{opt}}.}\simeq 0.5$. It may be interesting to compare the performance improvement by introducing the mixture reservoir with that by simply increasing the number of nodes which were reported by Rodan & Tino^36^. For the 1-step ahead prediction of the SantaFe data set, the comparison suggests a conjecture; *replacing a small number of nonlinear nodes with linear nodes* improves RC performance as effective as *doubling the number of nonlinear nodes*. See the Supplementary Information for a detailed comparative argument.

As future work, it is important to study the universality of the memory-nonlinearity trade-off and the effect of the mixture reservoir, i.e., to see if the results presented in this paper hold in other reservoirs, e.g. with different network topology, and for other tasks. Theoretically, it would be interesting to clarify the relationships between the quantities relating to the memory, i.e. the (maximal) conditional Lyapunov exponent, the linear memory capacity^20^ *MC*, and the mutual information *I*(*δ*_0_; *δ*_*n*_). These relationships could provide a strategy for determining the optimal reservoir parameters for its performance. To quantify the memory capacity of the mixture reservoir, it may be interesting to study the mutual information in the case of the mixture reservoir and how the mutual information changes with the mixture rate *p*. Moreover, it is an important future work to compare the mixture reservoir with other methods such as RC with random static projection (R^2^SP)^29–31^. One of applications of the idea of the mixture reservoir is to add an auxiliary linear feedback to the implementation of RC with delay feedback (i.e., adding linear virtual nodes), which could improve its performance remarkably.

In this work, we found that one of the characteristics of dynamical systems suitable for RC is the coexistence of both linear and nonlinear dynamics. This is a step toward uncovering a guiding principle of reservoir design for high-performance information processing, which is expected to provide an answer to the question stated in the introduction: for a given task, what characteristics of a dynamical system are crucial for information processing? Once revealed, such a guiding principle will enrich our knowledge of computer science, deepen our understanding of brain functions, and contribute to extending dynamical system theory.

## Electronic supplementary material

Supplementary Information
