# Supplementary material for: Reservoir Computing Beyond Memory-Nonlinearity Trade-off
Source: Sci Rep. 2017 Aug 31;7:10199. doi: 10.1038/s41598-017-10257-6 (PMC5579006; doi:10.1038/s41598-017-10257-6)
Supplement: Supplementary file 1 — Supplementary Information [file 41598_2017_10257_MOESM1_ESM.pdf]

Supplementary Information: Reservoir Computing Beyond Memory-Nonlinearity Trade-off

Masanobu Inubushi[a] and Kazuyuki Yoshimura[b]
a. NTT Communication Science Laboratories, NTT Corporation,
3-1, Morinosato Wakamiya Atsugi-shi, Kanagawa, 243-0198, Japan.
b. Department of Information and Electronics, Graduate School of Engineering,
Tottori University, 4-101 Koyama-Minami, Tottori 680-8552, Japan

August 3, 2017

1 Conditional Lyapunov spectrum

In the main text, Fig. 1 (b) shows the conditional Lyapunov spectrum of the echo state network for $g = 0.5, 1.0, 2.0$ with the fixed parameter $\epsilon = 0.5$. The conditional Lyapunov spectrum is calculated numerically by using the standard QR decomposition method[2]. When g is small (e.g. $g = 0.5$), the reservoir states seems to be in the neighborhood of the fixed point at the origin. Therefore, the conditional Lyapunov spectrum λ_i $(i = 1, \cdots, N)$ is close to the spectrum $\ln|\sigma_i(gJ)|$ $(i = 1, \cdots, N)$, where $\sigma_i(gJ)$ is an eigenvalue of the connection matrix gJ (the distribution of the eigenvalues is depicted in the inset of the Fig. 1 (b)). With increasing g, the Lyapunov spectrum λ_i becomes 'smooth' with respect to the index i (see the case of $g = 1.0$ in the Fig. 1 (b)). Moreover, the positive Lyapunov exponents appear when $g \gtrsim 1.5$, and the reservoir states go outside the neighborhood of the origin where the nonlinearity plays the dominant role. Correspondingly, the shape of the Lyapunov spectrum changes significantly (see the case when $g = 2.0$ in Figure. 1 (b)).

2 Relation between the linear and nonlinear reservoirs

As noted in the main text, the linear reservoir can be interpreted as $\epsilon \to 0$ limit of the nonlinear reservoir. To see this, we need to pay attention on the following two points. First, the performance of the linear reservoir does not depend on the parameter ϵ (> 0), since we can scale the variable as $\tilde{x} = x/\epsilon$ and eliminate the parameter ϵ from the evolution equation (1) and (2) in the

main text. Second, considering the Taylor expansion of the activation function ($\phi[a] = \tanh a \simeq a - a^3/3$) and the balance of the linear term (a) and the nonlinear term ($a^3/3$), the nonlinearity of the dynamics is negligible in the parameter region $\epsilon \ll cg^{-1}$ with a constant $c = O(1)$.

Let us consider that the minimal error by the linear reservoir for some task is attained at the parameters (g^*, ϵ^*), and we write the minimal error as $\min_{g,\epsilon} E_L = E_L(g^*, \epsilon^*)$. From the first point, $E_L(g^*, \epsilon^*) = E_L(g^*, \epsilon) \quad (\forall \epsilon > 0)$. For the parameter region $\epsilon \ll cg^{-1}$, the linear and nonlinear reservoirs show similar dynamics, and their performances are also similar; $E_L(g, \epsilon) \simeq E_{NL}(g, \epsilon)$, where E_{NL} denotes error by the nonlinear reservoir. Therefore, taking $g = g^*$ and considering the parameter region $\epsilon \ll c{g^*}^{-1}$, we obtain $\min_{g,\epsilon} E_L \simeq E_{NL}(g^*, \epsilon)$. Hence, we conclude that the optimal performance by the linear reservoir can be always approximated by the nonlinear reservoir taking sufficiently small ϵ. In this sense, the linear reservoir can be interpreted as $\epsilon \to 0$ limit of the nonlinear reservoir.

From the numerical results, we see the linear reservoir significantly outperforms the nonlinear reservoir for some task parameters (for instance, Figure 2 (b) in the main text). However, from the above arguments, the performance of the linear reservoir can be always approximated by that of the nonlinear reservoir arbitrarily well by using sufficiently small ϵ. Therefore, theoretically, the performance of the nonlinear reservoir should be better than or almost the same as that of the linear reservoir. The reason why there are some task parameters in the numerical results where the linear reservoir significantly outperforms the nonlinear reservoir is that we use finite step sizes Δg and $\Delta \epsilon$ for the optimization of the control parameters in the numerical experiments.

We give a more detailed description of the reason. As mentioned above, $E_L(g^*, \epsilon)$ is constant for ϵ (> 0). And, in the case that the linear reservoir outperforms the nonlinear reservoir, $\Delta E(g^*, \epsilon) > 0$ and $\Delta E(g^*, \epsilon) \to 0$ ($\epsilon \to 0$) where ΔE represents the difference in the errors $\Delta E(g^*, \epsilon) := E_{NL}(g^*, \epsilon) - E_L(g^*, \epsilon)$ for some fixed parameter $g = g^*$. In the numerical experiments, because of the finiteness of the step size $\Delta \epsilon$, the difference is always positive $\Delta E(g^*, \Delta \epsilon) > 0$. Hence, due to the finiteness of the step size, we can see that the linear reservoir can outperform the nonlinear reservoir significantly for some tasks in the numerical results.

The difference $\Delta E(g^*, \Delta \epsilon) > 0$ decreases with decreasing step size $\Delta \epsilon$ in the case that the linear reservoir outperforms the nonlinear reservoir. Indeed, this behavior can be observed in Figure b in this Supplementary Information. For instance, in Figure b (c-1) and (c-2), the linear reservoir outperforms the nonlinear reservoir. The difference $\Delta E(g^*, \Delta \epsilon) > 0$ decreases by replacing P_{small} (the rougher step sizes) with P_{large} (the finer step sizes). Although the numerical results such as the value ΔE may depend on the step sizes quantitatively, we check and show that the main results in the main text are insensitive to the choice of the step sizes in Section 5 in this Supplementary Information in detail.

3 Nonlinearity degrades memory for general dimensional case

In the main text, using the variational equation and the mutual information, we gave a theoretical interpretation of the nonlinearity-memory trade-off; in particular, we showed how nonlinear dynamics degrades memory. Although we discussed the trade-off for the $N = 1$ case in the main text, the interpretation also holds for general dimensional case ($N \geq 1$) simply by replacing the equation (5) with

$$\delta_n = g^n DT^n \left[x(t_0), \{s(t_0 + i)\}_{i=0}^{n-1}\right] \delta_0. \qquad (*)$$

Here, δ_n ($n \geq 1$) denotes N-dimensional tangent vector and

$$DT^n \left[x(t_0), \{s(t_0 + i)\}_{i=0}^{n-1}\right]$$
$$:= DT(x(t_0 + n - 1), s(t_0 + n - 1)) \cdots DT(x(t_0 + 1), s(t_0 + 1)) \cdot DT(x(t_0), s(t_0)),$$

where $DT(x(t), s(t))$ is the Jacobian matrix defined by the equation (4) and $\cdot$ represents the matrix multiplication. As discussed in the main text, the strong relation between δ_0 and δ_n indicates a large memory capacity of the reservoir. For the nonlinear reservoir, the relation is given by the linear transformation $(*)$. This relation depends on the sequences of the states and input signals via the Jacobian matrix DT^n, which are 'noise' in view of preserving information of δ_0. On the other hand, the relation for the linear reservoir is given by the linear transformation which does *not* depend on the sequences, i.e., $\delta_n = (gJ)^n \delta_0$. Apparently, if J is invertible, we can reconstruct δ_0 from the data δ_n without any additional information, i.e, $\delta_0 = (gJ)^{-n} \delta_n$. Therefore, it can be interpreted, for the general dimensional case, that the introducing nonlinearity in reservoir dynamics degrades memory.

4 Enlarged view of Figure 3 in the main text

In Figure 3 in the main text, the error values around $p = 0$ may be difficult to see, and thus, we here show the enlarged view of the figure in Figure a.

5 Insensitivity of numerical results to choice of parameter searching region

In the main text, we evaluated the RC performance by the error defined as follows: $\mathcal{E} := \min_{(g,\epsilon)\in P} E(w^*|g, \epsilon)$, where $P := \{(g, \epsilon)|g \in [0.1, 3.0], \epsilon \in [0.2, 6.0]$ with step size $\Delta g = 0.1, \Delta\epsilon = 0.2\}$ is the parameter searching region.

We here show the numerical results are insensitive to the choice of the parameter searching region, by changing the area and the step size of P. To this end, we introduce two parameter searching region P_{small} and P_{large}:

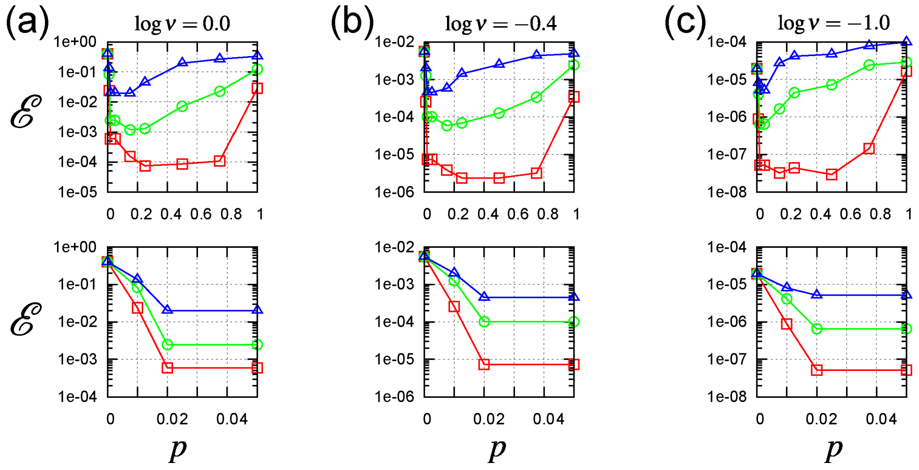

Figure a.: **Enlarged view of Figure 3.** The upper panels are the same figures shown in Figure 3 in the main text. The lower panels are the corresponding enlarged view ($0 \leq p \leq 0.05$) of the upper panels.

- $P_{small} := \{(g, \epsilon)|g \in [0.5, 2.0], \epsilon \in [0.5, 3.0]$ with $\Delta g = 0.5, \Delta\epsilon = 0.5\}$,
- $P_{large} := \{(g, \epsilon)|g \in [0.05, 5.0], \epsilon \in [0.1, 8.0]$ with step size $\Delta g = 0.05, \Delta\epsilon = 0.1\}$.

P_{small} is smaller area and rougher step size than P, and P_{large} is larger area and finer step size than P. In Figure b, we show the errors $\mathcal{E}$ calculated with the parameter searching regions P_{small} (Figure b (a-1), (b-1), and (c-1)) and P_{large} (Figure b (a-2), (b-2), and (c-2)), The figures (a-c) corresponds to the figures (a-c) in Figure 3 in the main text which calculated with P. While the error values are increased by using P_{small} and decreased by using P_{large} compared with those by using P as expected, we can see that, for $0 < p < 1$, $\mathcal{E}(p) < \mathcal{E}(p = 0)$ or $\mathcal{E}(p = 1)$ in each case. Therefore, we conclude that one of our main claims, i.e., introducing the mixture reservoir suppresses errors, is insensitive to the choice of the parameter searching region qualitatively.

6 Performance improvements by introducing the mixture reservoir vs. by increasing the number of nodes.

As mentioned in the main text, it may be interesting to compare the performance improvement by replacing nonlinear nodes with linear nodes (i.e., using the mixture reservoir) with a fixed number of nodes ($N = 100$) with the performance improvement by increasing the number of nodes N of the nonlinear reservoir ($p = 1$) which were reported by Rodan & Tino [1] in detail.

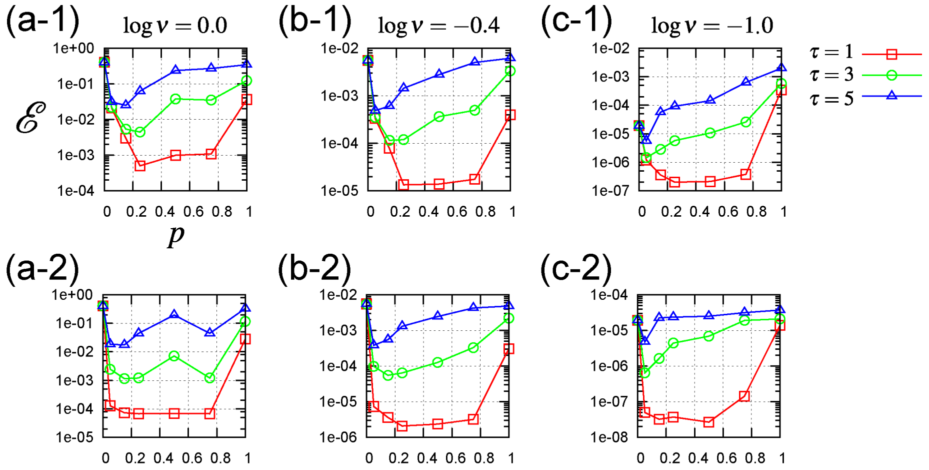

Figure b.: **Insensitivity of numerical results to choice of parameter searching region.** The errors $\mathcal{E}$ calculated with the parameter searching regions P_{small} ((a-1), (b-1), and (c-1)) and P_{large} ((a-2), (b-2), and (c-2)). The figures (a-c) corresponds to the figures (a-c) in Figure 3 in the main text which calculated with P.

They reported the performances of the echo state network model with the similar settings to our nonlinear reservoir ($p = 1$) for both the SantaFe and the NARMA task, and provided the error values in detail (Fig. 3, 4, and TABLE IV in [1]). For the 1-step ahead prediction of the SantaFe data set, the normalized mean squared errors were reported as 0.0125 with $N = 100$ and 0.00819 with $N = 200$ in TABLE IV in [1]. On the other hand, in our case with fixed $N = 100$, the normalized mean square errors were 0.00382 with $p = 1$ and 0.00245 with $p = 0.95$ shown in Figure 5. The error value by our nonlinear reservoir ($p = 1$) is smaller than that reported in [1], which may be due to disagreements in the settings of the numerical experiments. In particular, while we used the control parameters (g, ϵ) and optimized them, they used different control parameters and did not optimize them.

Instead, here we compare the error suppression rates R; increasing the number of nodes results in $R = E(N = 200)/E(N = 100) \simeq 0.66$, while replacing nonlinear nodes for linear nodes results in $R = E(p = 0.95)/E(p = 1.0) \simeq 0.64$. This suggests a conjecture; *replacing a small number of nonlinear nodes for linear nodes* improves RC performance as effective as *doubling the number of nonlinear nodes.*

However, this conclusion is not valid for the NARMA10 task, i.e., the effectivity of the above replacement (mixture reservoir) strongly depends on the task. For the NARMA10 task, while the error value 0.0956 with $N = 100$ can be decreased to 0.0425 with $N = 200$ reported in TABLE IV[1], i.e., $R \simeq 0.44$, the

error values are almost same for all mixture rates p; the error value 0.0892 with $p = 1$ and 0.0823 with $p = 0.5$, i.e., $R \simeq 0.92$. Although the above conclusions should be verified based on a systematic comparative study, it is beyond the scope of this paper and a future work.

References

[1] Rodan, A & Tino, P. Minimum Complexity Echo State Network. *IEEE transaction on neural networks*, 22, 1 (2011)

[2] Shimada, I., & Nagashima, T. A Numerical Approach to Ergodic Problem of Dissipative Dynamical Systems. *Prog Theor Phys.* 61 (6): 1605-1616 (1979).
